# Supplementary material for: Parvalbumin-expressing basal forebrain neurons mediate learning from negative experience
Source: Nat Commun. 2024 Jun 7;15:4768. doi: 10.1038/s41467-024-48755-7 (PMC11161511; doi:10.1038/s41467-024-48755-7)
Supplement: Supplementary file 1 — Supplementary Figs. [file 41467_2024_48755_MOESM1_ESM.pdf]

Parvalbumin-expressing basal forebrain neurons mediate learning from negative experience

Hegedüs, Király & Schlingloff et al.

Supplementary Information

Supplementary Figures

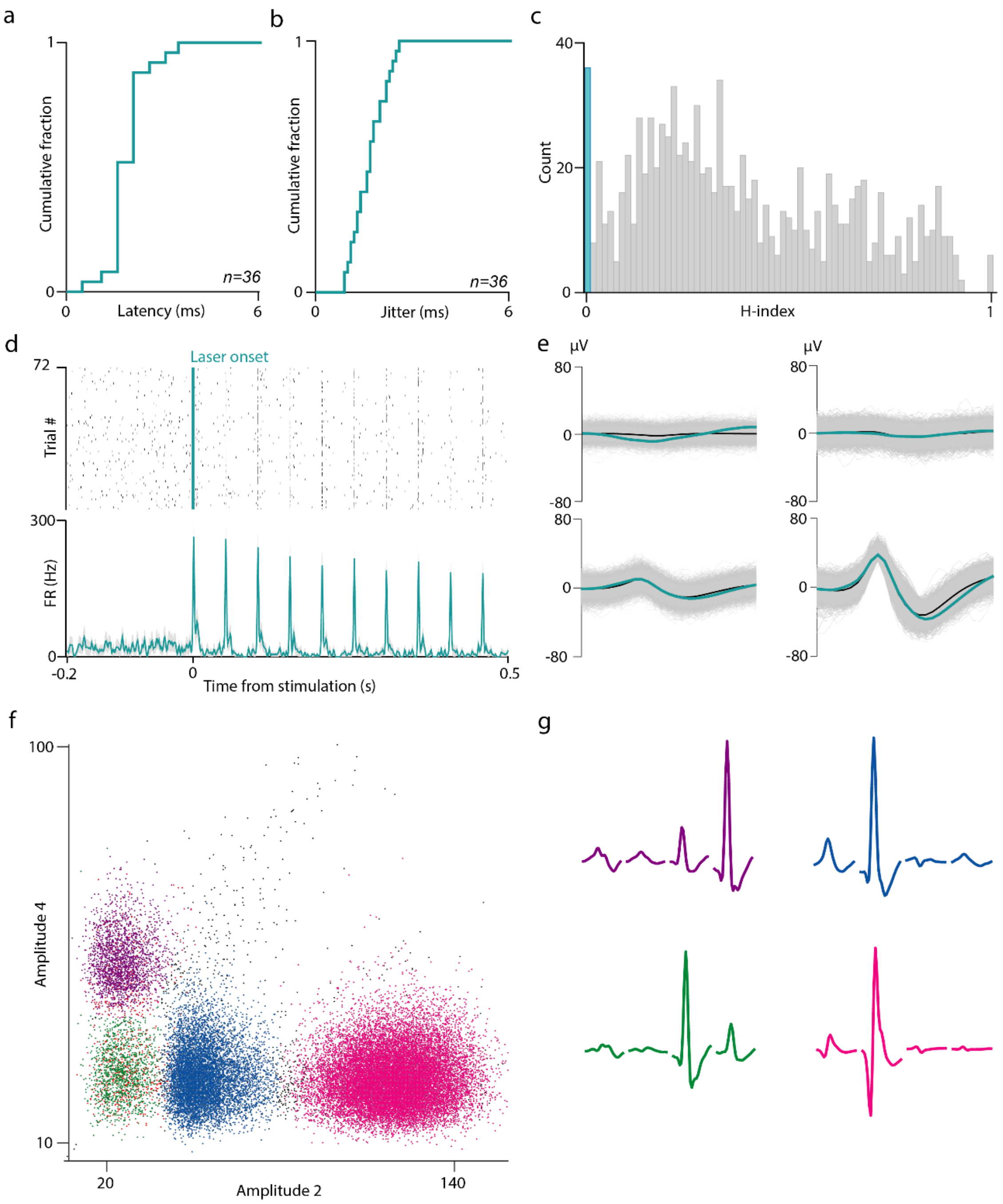

Figure S1. Optogenetic tagging of HDB BFPVNs. a, Cumulative histogram of the peak response latency of BFPVNs after optogenetic stimulation (n = 36). **b,** Cumulative histogram of the jitter of BFPVN spike responses after optogenetic stimulation (n = 36). **c,** Distribution of the significance values of the SALT statistical test (H-index) for all recorded neurons (blue, p < 0.01, tagged BFPVNs; grey, p > 0.01, untagged neurons). **d,** Example spike raster and PETH of an optogenetically tagged BFPVN responding to 20 Hz blue laser light stimulation. **e,**

Average spike waveform of the same BFPVN on the four tetrode channels (blue, average light-evoked spikes; black, average spontaneous spikes; grey, all spikes). **f,** Spike clusters plotted in feature space from an example recording session. **g,** Average spike waveforms of the recorded neurons on each tetrode channel from the same session.

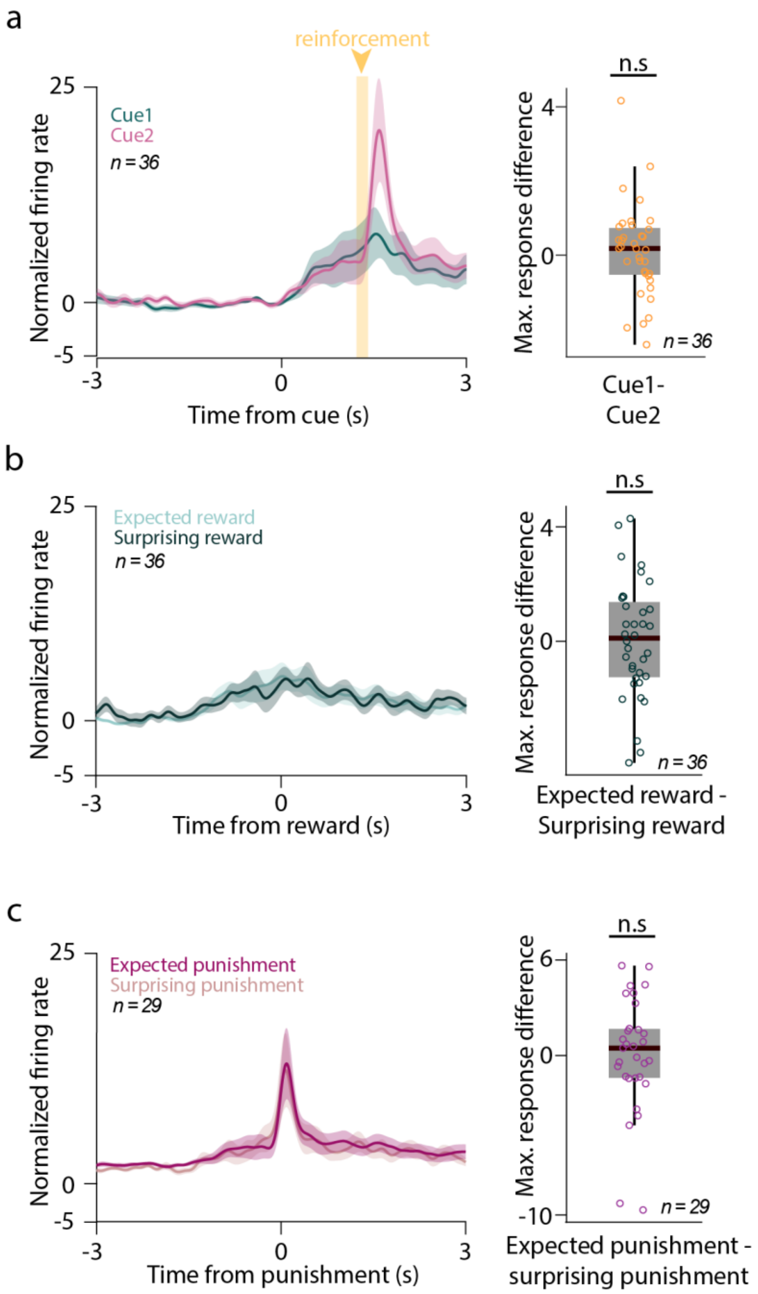

Figure S2. HDB BFPVNs are not modulated by outcome expectation. a, Left, average PETH of BFPVNs aligned to cue onset (n = 36). Right, difference of peak response to Cue 1 and Cue 2 (in a 0-0.5s time window from cue onset). n.s., $p > 0.05$, $p = 0.6151$, two-sided Wilcoxon signed-rank test. **b,** Left, average PETH of BFPVNs aligned to expected and surprising reward (n = 36). Right, difference of peak response to expected and surprising reward. n.s., $p > 0.05$, $p = 0.8628$, two-sided Wilcoxon signed-rank test. **c,** Left, average PETH of BFPVNs aligned to expected and surprising punishment (n = 29, 7 neurons excluded from this analysis because there were only 5 or less surprising punishments in the session). Right, difference of peak response to expected and surprising punishment. n.s., $p > 0.05$, $p = 0.5566$, two-sided Wilcoxon signed-rank test. Source data are provided as a Source Data file.

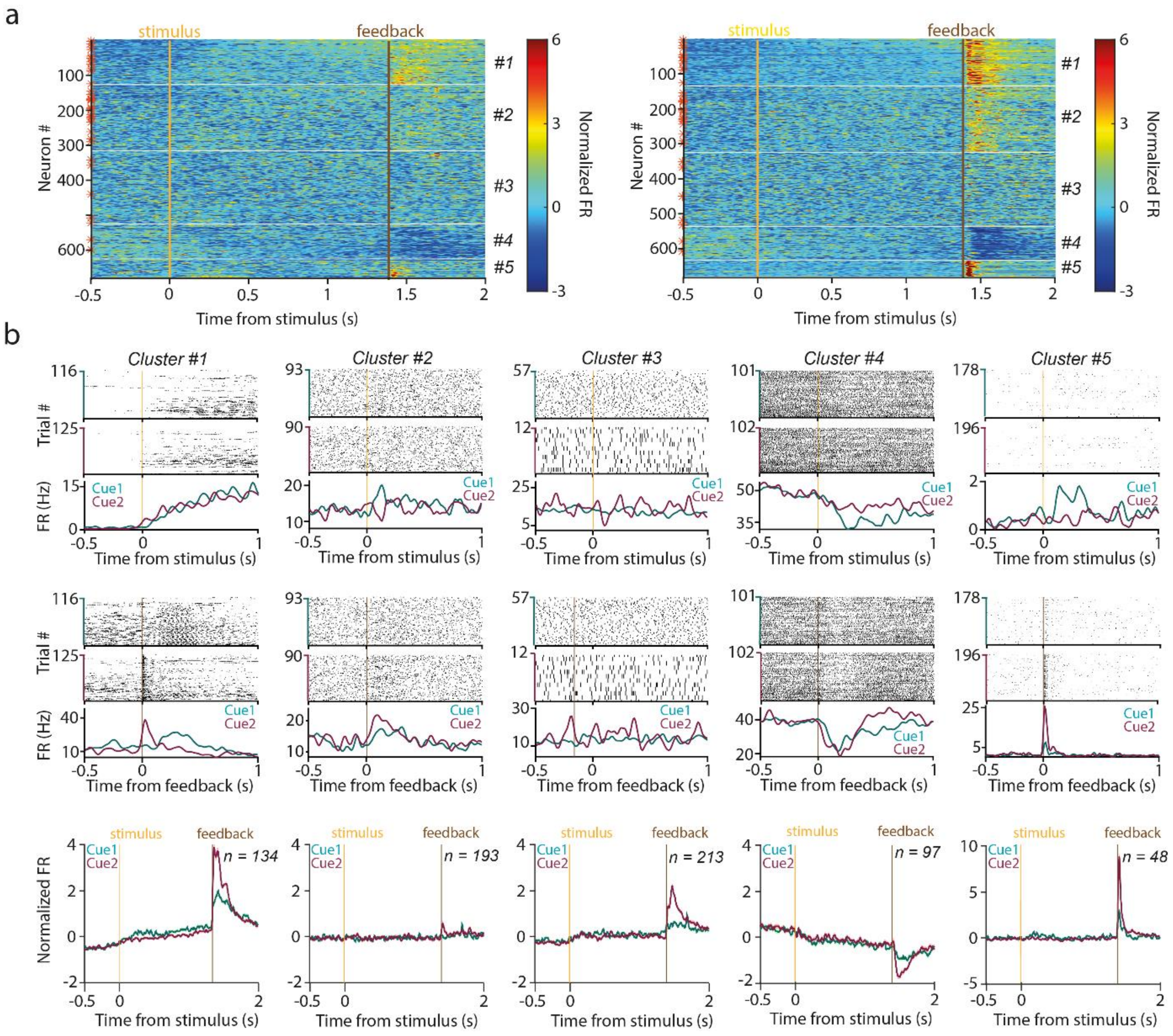

Figure S3. K-means clustering of BF neuronal responses reveals groups of neurons with distinct firing patterns. a, Color-coded, Z-scored PETHs of all neurons aligned to Cue 1 (left) and Cue 2 (right; n = 685). Red asterisks indicate tagged BFPVNs. The clusters were ordered according to percentage of tagged neurons. **b,** Top and middle, PETH of example neurons aligned to stimulus (top) and reinforcement (middle). Bottom, Average, Z-scored PETH of each cluster.

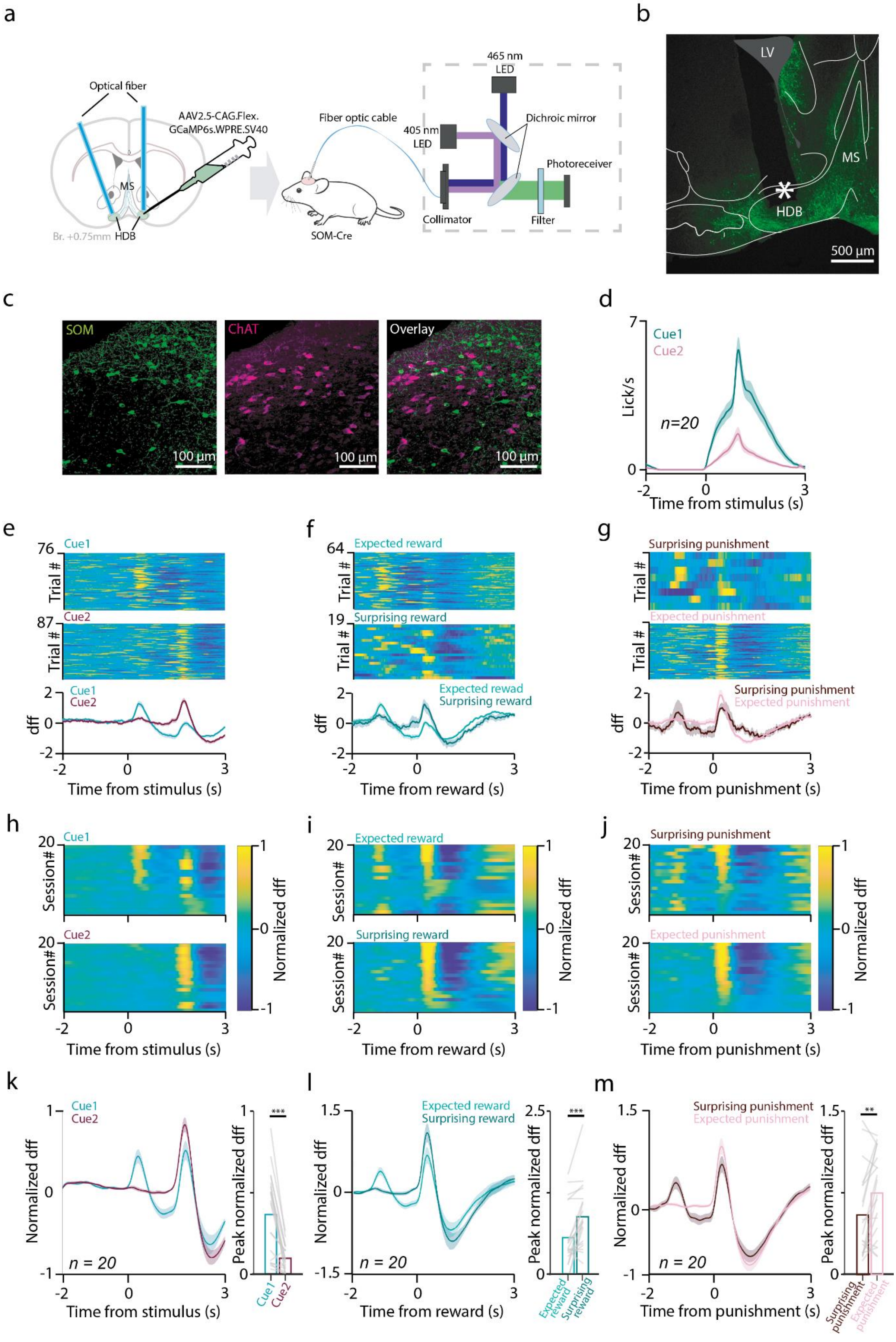
a
Optical fiber
AAV2.5-CAG.Flex.
GCaMP6s.WPRE.SV40
MS
Br. +0.75mm HDB
Fiber optic cable
SOM-Cre
465 nm
LED
405 nm
LED
Dichroic mirror
Photoreceiver
Collimator
Filter
b
LV
MS
HDB
500 µm
c
SOM
ChAT
Overlay
100 µm
d
Cue1
Cue2
Lick/s
n=20
Time from stimulus (s)
e
Cue1
Cue2
Trial #
dff
Time from stimulus (s)
f
Expected reward
Surprising reward
Expected rewad
Trial #
dff
Time from reward (s)
g
Surprising punishment
Expected punishment
Trial #
dff
Time from punishment (s)
h
Cue1
Cue2
Session#
Normalized dff
Time from stimulus (s)
i
Expected reward
Surprising reward
Session#
Normalized dff
Time from reward (s)
j
Surprising punishment
Expected punishment
Session#
Normalized dff
Time from punishment (s)
k
Cue1
Cue2
Normalized dff
Peak normalized dff
n = 20
***
Time from stimulus (s)
l
Expected reward
Surprising reward
Normalized dff
Peak normalized dff
n = 20
***
Time from reward (s)
m
Surprising punishment
Expected punishment
Normalized dff
Peak normalized dff
n = 20
**
Time from punishment (s)

Figure S4. BFSOMNs respond to reward, punishment, and reward-predictive auditory cues. a, Schematic diagram of bulk calcium measurements of BFSOMNs. Created using Tyler, E., & Kravitz, L. (2020). mouse, Zenodo, https://doi.org/10.5281/zenodo.3925901, under Creative Commons 4.0 license (https://creativecommons.org/licenses/by/4.0/). The original image was modified by adding illustrations of optic cable and photometry recording system. **b**, Fluoromicrograph of an optical fiber track (green, GCaMP6s; asterisk, tip of the optical fiber). **c**, Immunohistochemical staining of the HDB shows no overlap between BFSOMNs and BFCNs (green, SOM; magenta, ChAT; 8 out of 392 SOM cells (~ 2%) expressed ChAT, n = 5 animals). **d,** SOM-Cre mice have learned the task indicated by higher anticipatory lick rate to the reward predicting cue. **e-g,** PETHs of bulk-calcium recording of BFSOMNs aligned to cue (left), reward (middle) and punishment (right) in an example session (top, trial-by-trial data; bottom, session average). **h-j,** Color-coded PETH showing all recorded training sessions where animals have acquired the task contingencies, aligned to cue (left), reward (middle) and punishment (right). **k-m,** Average PETHs of BFSOMN response during the task, aligned to cue (left), reward (middle) and punishment (right). PETHs were smoothed with a Gaussian kernel (width, 100 ms). Bar plots represent the mean of the peak response distribution. Errorshades on all PETHs indicate SEM. Two-sided Wilcoxon signed-rank test; **, $p \leq 0.01$; ***, $p \leq 0.001$. Cue responses, p = 0.000189; reward responses, p = 0.000681; punishment responses, p = 0.01. Source data are provided as a Source Data file.

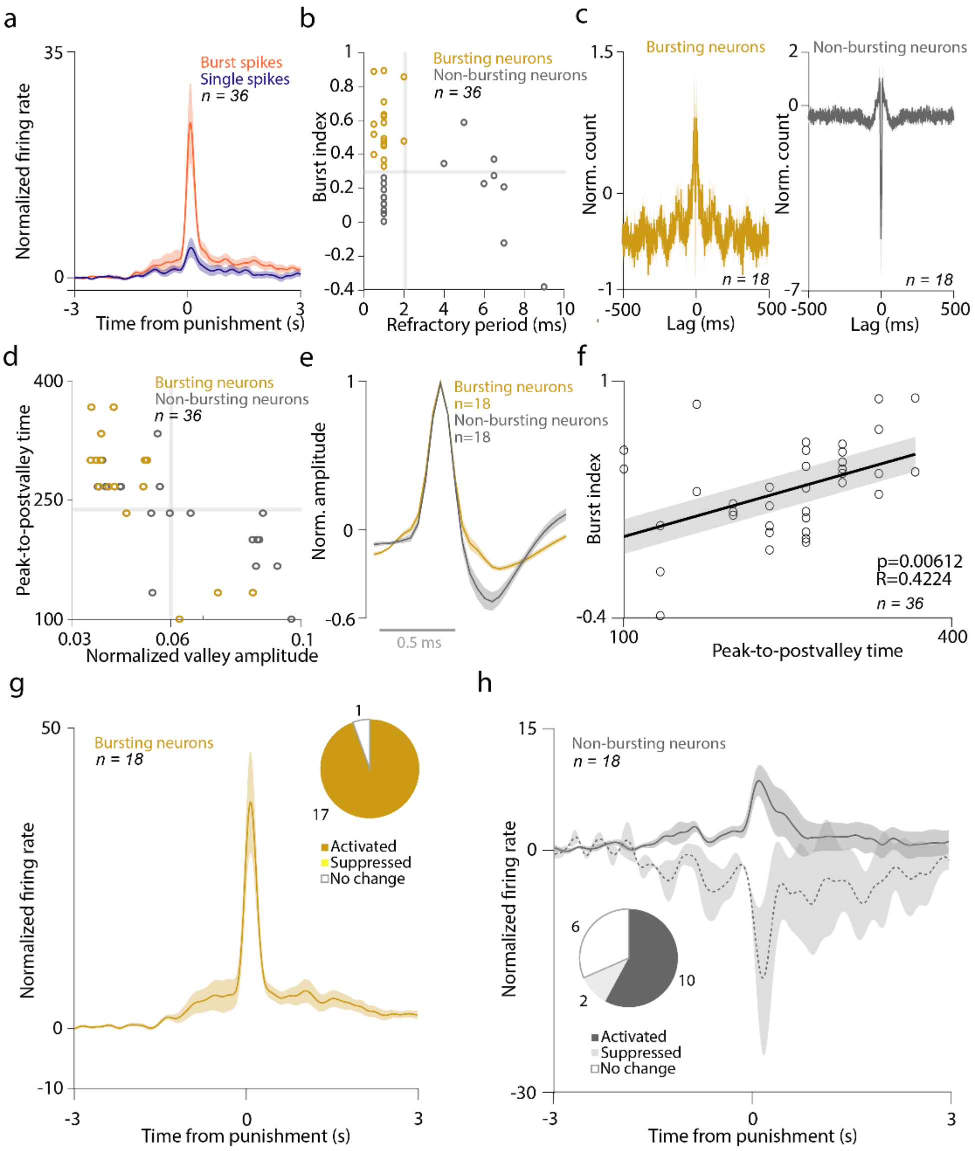

Figure S5. Electrophysiological properties of HDB BFPVNs. a, Average PETH of burst spikes and single spikes aligned to punishment onset (n = 36; errorshade, SEM). **b,** BFPVNs were partitioned based on burst index and refractory period. Neurons with high (> 0.3) burst index and short (< 2 ms) refractory period were considered as bursting neurons. Neurons with low burst index and/or long refractory period were considered non-bursting. **c,** Average autocorrelogram of bursting (left) and non-bursting (right) neurons (errorshade, SEM). **d,** Spike shape features of BFPVNs (peak-to-post-valley time and post-valley magnitude, normalized to the integral). Most bursting neurons had smaller valley and longer peak-to-post-valley time. **e,** Average spike shape of bursting and non-bursting neurons (errorshade, SEM). **f,** Correlation of burst index and peak-to-post-valley time. **g,** Average PETH of bursting BFPVNs aligned to punishment (errorshade, SEM). Pie chart showing activation, suppression or no response to

punishment of bursting BFPVNs (n = 18). **h,** Average PETH of non-bursting BFPVNs aligned to punishment (errorshade, SEM). Pie chart showing activation, suppression, or no response to punishment of non-bursting BFPVNs (n = 18). Source data are provided as a Source Data file.

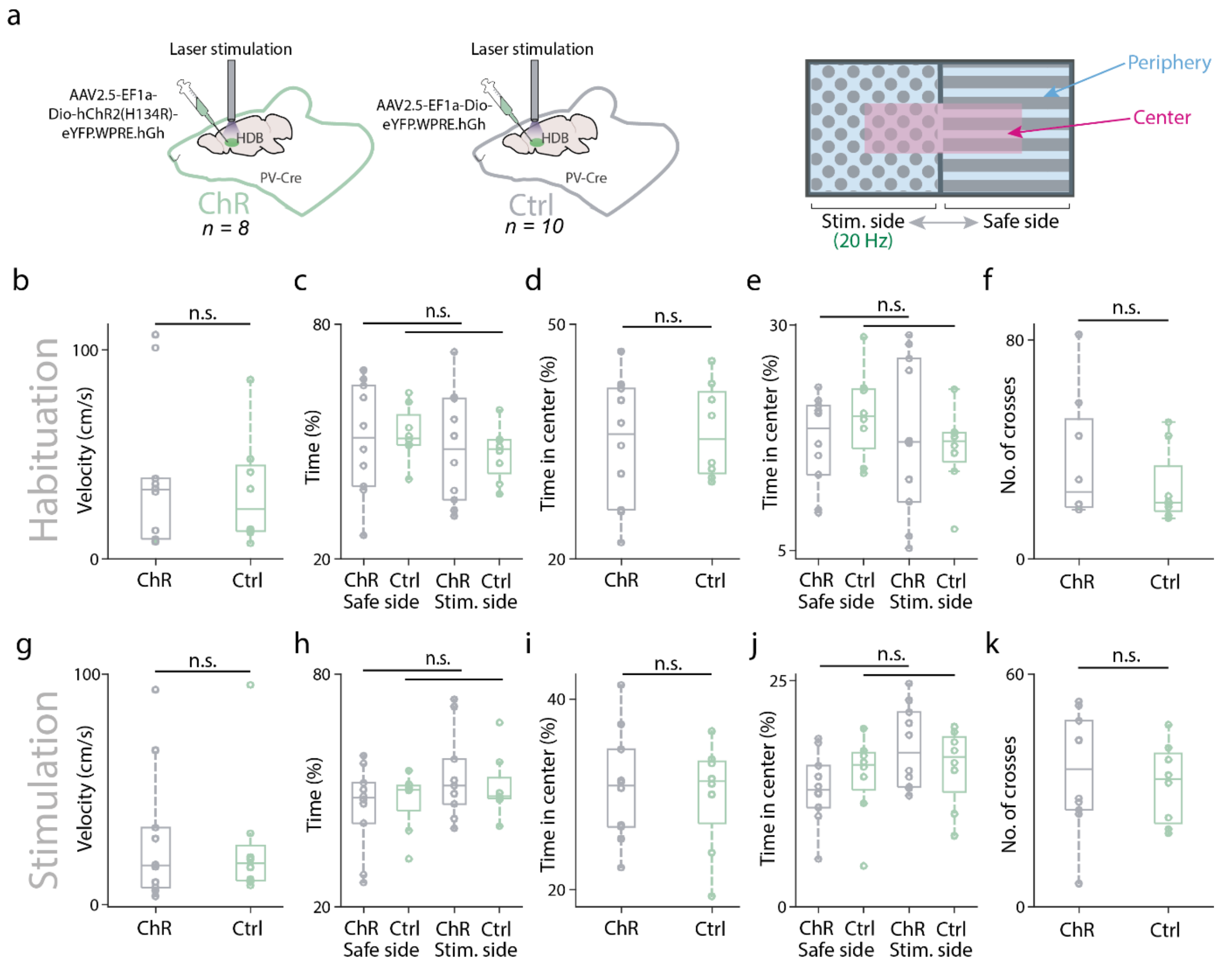

Fig S6. Conditioned place aversion. a, Schematic illustration of the experiment (for further details, see Methods). ChR, channelrhodopsin; Ctrl, control; Stim. side, stimulated side. Created using Kennedy, A. (2020) Mouse brain silhouette, Zenodo, https://doi.org/10.5281/zenodo.3925919, under Creative Commons 4.0 license (https://creativecommons.org/licenses/by/4.0/). The original image was modified by adding illustrations of a syringe and an optic fiber as well as modifying colors. **b,** Velocity of channelrhodopsin-expressing (ChR, n = 8) and control (Ctrl, n = 10) animals during habituation (n.s., $p > 0.05$, $p = 0.4557$, Mann-Whitney U-test). Box plots indicate median and interquartile range; whiskers indicate the non-outlier range in all graphs in this figure. **c,** Proportion of time spent on the safe (non-stimulated) and stimulated side during habituation (n.s., $p > 0.05$, $p = 0.9591$ and $p = 0.6454$, Mann-Whitney U-test). **d,** Overall proportion of time spent in the center area during habituation (n.s., $p > 0.05$, $p = 0.8673$, Mann-Whitney U-test). **e,** Proportion of time spent in center area on the stimulated and safe sides during habituation (n.s., $p > 0.05$, $p = 0.2134$ and $p = 0.7643$, Mann-Whitney U-test). **f,** Number of side crosses during habituation (n.s., $p > 0.05$, $p = 0.4538$, Mann-Whitney U-test). **g-k,** Same as in b-f, but during the

stimulation phase (n.s., $p > 0.05$, $p = 0.6334$, $p = 0.7985$, $p = 0.995$, $p = 0.6734$, $p = 0.4352$, $p = 0.2452$, $p = 0.3955$, respectively, Mann-Whitney U-test). Source data are provided as a Source Data file.

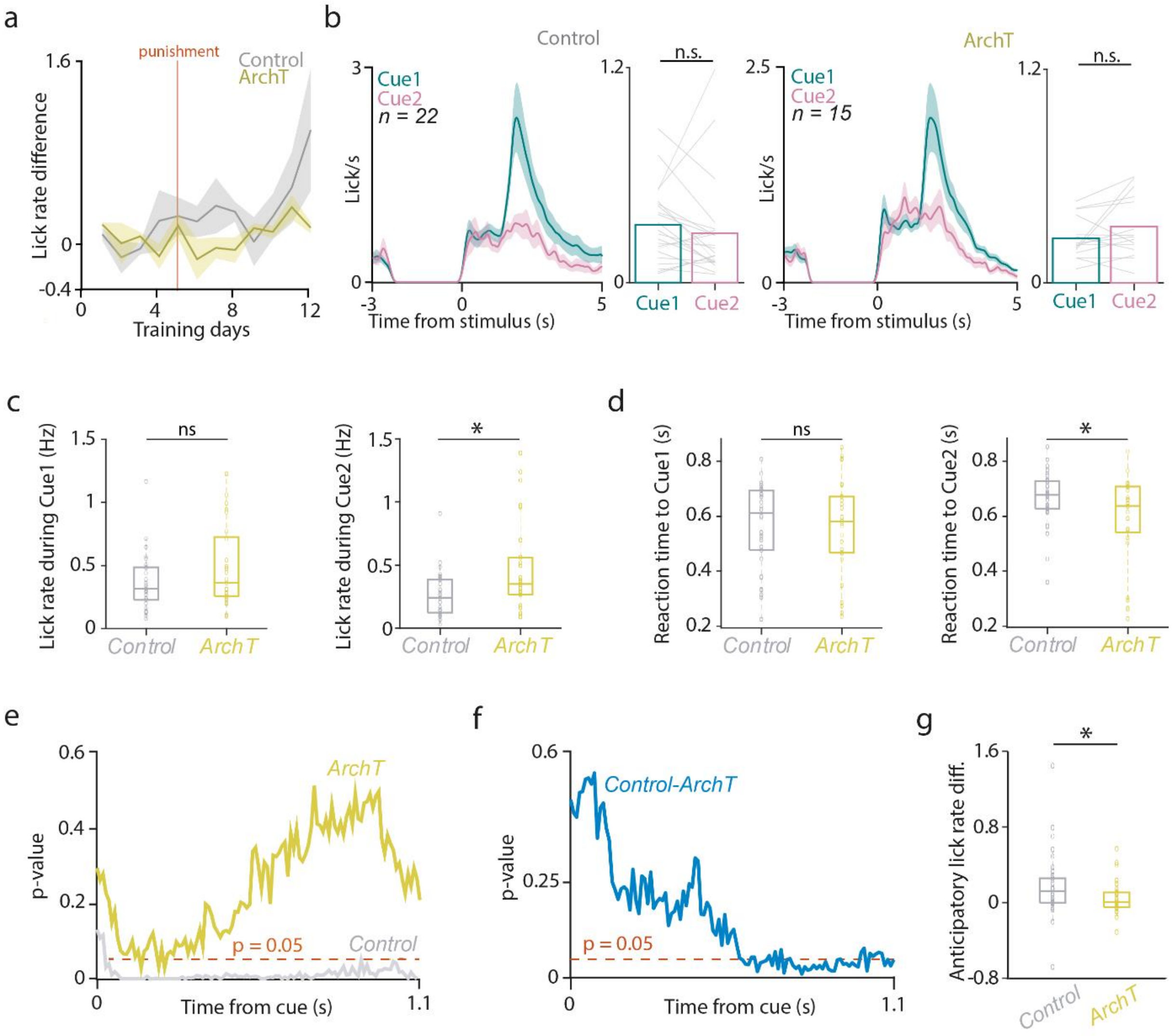

Fig S7. Differential learning in Control and ArchT-inhibited mice. a Learning curve of Control and ArchT animals (anticipatory lick rate difference plotted as a function of training days; errorshade, SEM). **b**, PETH and bar graph showing no anticipatory lick rate difference between Control and ArchT mice at an earlier training stage (before introducing punishment; two-sided Wilcoxon signed-rank test; p = 0.101 for Control and p = 0.173 for ArchT; n.s., p > 0.05; errorshade, SEM). **c**, Lick rate during Cue1 and Cue2 presentation in Control and ArchT mice. *, p < 0.05, p = 0.373 for Cue1, p = 0.013 for Cue2, two-sided Mann-Whitney U-test. Box-whisker plots indicate median, interquartile range and non-outlier range. **d**, Reaction time to Cue1 and Cue2 in Control and ArchT mice. *, p < 0.05, p = 0.715 for Cue1, p = 0.0128 for Cue2, two-sided Mann-Whitney U-test. Box-whisker plots indicate median, interquartile range and non-outlier range. **e**, Statistical significance of cue-specific anticipatory lick rate difference as a function of time from cue onset (ROC analysis, see Methods). **f,** Statistical significance of the cue-related anticipatory lick rate difference between the ArchT and the control group as a

function of time from cue onset (ROC analysis, see Methods). The difference reached statistical significance around the middle of the response window due to large variability in reaction times. **g,** Anticipatory lick rate difference in control (left) and ArchT (right) groups in a 0.6-1.1 s window from cue onset. *, $p < 0.05$, $p = 0.03669$, two-sided Mann-Whitney U-test**.** Box-whisker plot indicates median, interquartile range and non-outlier range. Source data are provided as a Source Data file.

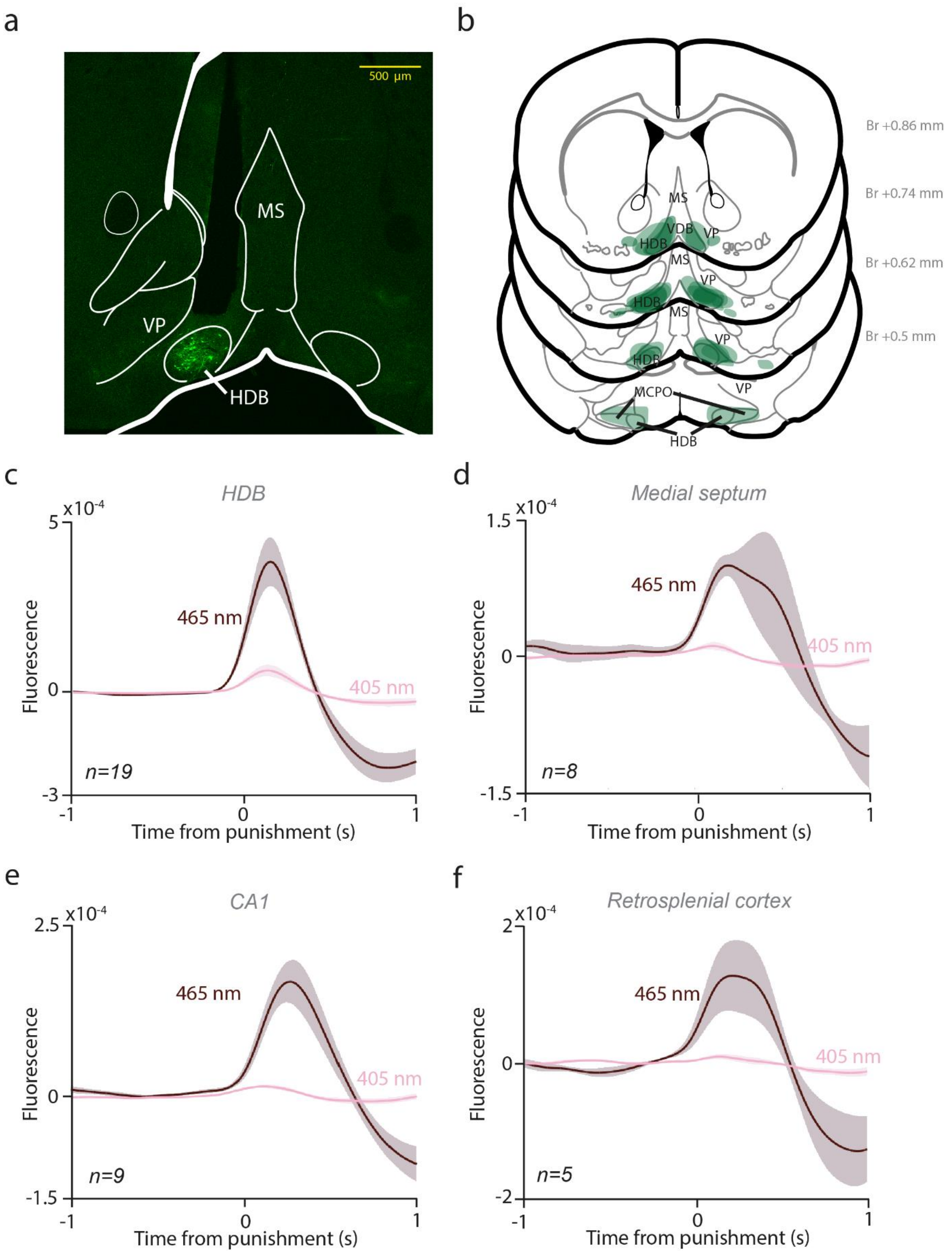

Fig S8. Track reconstruction and isosbestic channels of fiber photometry recordings. a, Representative fluoromicrograph of a GCaMP6s injection in a PV-Cre mouse (repeated in n = 12 mice). **b**, Reconstruction of all injection sites in the HDB. **c,** PETH of 465 nm and 405 nm wavelength fluorescent signals aligned to punishment, recorded in the HDB (n = 19 sessions;

errorshade, SEM). **d,** PETH of 465 nm and 405 nm wavelength fluorescent signals aligned to punishment, recorded in the MS (n = 8 sessions; errorshade, SEM). **e,** PETH of 465 nm and 405 nm wavelength fluorescent signals aligned to punishment, recorded in the CA1 hippocampus (n = 9 sessions; errorshade, SEM). **f,** PETH of 465 nm and 405 nm wavelength fluorescent signals aligned to punishment, recorded in the RSC (n = 5 sessions; errorshade, SEM). PETHs were smoothed with a Gaussian kernel (width, 100 ms).

Brain areas	Fraction of total input neurons
Lateral hypothalamus	33.09
Lateral septum	13.81
intermediate part	10.18
dorsal part	2.89
ventral part	0.74
Medial septum	10.77
Vertical limb of the diagonal band	6.37
Preoptic area	7.62
lateral preoptic area	5.96
medial preoptic area	1.66
Horizontal limb of the diagonal band	5.81
Median raphe region	3.93
paramedian raphe nucleus	2.52
median raphe nucleus	1.41
Ventral pallidum	3.47
Nucelus accumbens	3.46
shell part	2.23
core part	1.23
Posterior hypothalamic nucleus	2.59
Medial amygdaloid nucleus	2.04
Laterodorsal tegmental nucleus	1.26
Magnocellular nucl. of the lat. hypothalamus	1.23
Septohippocampal nucleus	1.14
Gigantocellular reticular nucleus	1.01
Orbital cortex	0.90
CA3 stratum oriens	0.78
Nucleus incertus	0.72
TOTAL	100%

Table S1. Fraction of total inputs to HDB BFPVNs.

Antigen	Host	Dilution	Source	Catalog number	Specificity	Characterized in
eGFP	Chicken	1:2000	Thermo Fisher Scientific	A10262	No staining in mice not injected with eGFP-expressing virus.	Information of the distributor.
mCherry	Chicken	1:1000	Abcam	ab205402	Validated for WB, ICC/IF. Positive control: Lysate of HEK293 cells transfected with pFin-EF1-mCherry vector, HEK293 cells transfected with pFin-EF1-mCherry vector.	Information of the distributor.
mCherry	Rabbit	1:2000	BioVision	5993-100	No staining in mice not injected with mCherry-expressing virus.	Information of the distributor.
vGluT3	Guinea pig	1:1000	Frontier Institute	AF510321	Immunoblot detects a single protein band at 60-62 kDa.[1] Antibody to VGLUT3, (1 µg/mL) were absorbed to the whole fusion protein (30 µg/mL), which eliminated all labelling, indicating specificity.[2]	[1]Information of the distributor. [2] PMID: 14984406
RFP	Rat	1:2000	Chromotek	5F8	No staining in mice not injected with mCherry-expressing virus.	Information of the distributor.
5-HT	Rabbit	1:10000	Immunostar	20080	KO verified.	[104]
Chat	rabbit	1:1000 or 1:500	Synaptic Systems	297013	Specific for rat and mouse Chat.	Information of the distributor.
SOM	rabbit	1:500	Origene	AP-33464SU-N	Specificity: Recognizes Somatostatin-14	PMID:37205047
Chat	goat	1:1000	Merck	AB-144P	Specific for Chat in mouse among other species.	Information of the distributor.
PV	mouse	1:2000	Swant	PV235	Reacts specifically with parvalbumin in tissue originating from human, monkey, rabbit, rat, mouse, chicken and fish.	Information of the distributor.
PV	rabbit	1:1000	Swant	PV27	KO verified.	Information of the distributor.
CR	rabbit	1:10000	Swant	7697	KO verified.	Information of the distributor.

Table S2. Primary antibodies used in immunhistochemical experiments.

Raised in	Raised against	Conjugated with	Dilution	Source	Catalog number
Goat	Rabbit	Alexa 405	1:500	Invitrogen	A31556
Donkey	Chicken	Alexa 488	1:1000	Jackson Immunoresearch	703-545-155
Donkey	Rabbit	Alexa 594	1:500	Thermo Fisher Scientific	A21207
Goat	Chicken	Alexa 594	1:500	Abcam	Ab150172
Donkey	Guinea pig	Alexa 647	1:500	Jackson Immunoresearch	706-605-148
Donkey	Mouse	Alexa 647	1:500	Jackson Immunoresearch	715-605-150
Donkey	Rat	Alexa 594	1:500	Thermo Fisher Scientific	A21209
Donkey	Rabbit	Alexa 488	1:1000	Thermo Fisher Scientific	A21206
Donkey	Chicken	Biotin-SP	1:1000	Jackson Immunoresearch	703-065-155
Donkey	Goat	Alexa 594	1:1000	Thermo Fisher Scientific	A-11058

Table S3. Secondary antibodies used in immunohistochemical experiments.

Cellid	Baseline firing rate (Hz)	Peak firing rate after punishment (Hz)	Peak latency (ms)
'HDB17_170720a_4.2'	13.16	29.38	119
'HDB17_170723a_7.1'	11.14	22.89	20
'HDB17_170724a_7.2'	10.10	27.45	85
'HDB17_170725a_5.2'	21.93	47.88	91
'HDB17_170805a_5.1'	20.47	60.61	105
'HDB17_170807a_5.2'	18.24	34.08	158
'HDB17_170810a_3.1'	30.95	48.78	89
'HDB17_170810a_5.1'	26.10	89.49	83
'HDB17_170811a_3.2'	26.15	43.48	74
'HDB17_170812a_4.1'	12.48	21.06	73
'HDB17_170904a_4.2'	20.14	104.48	87
'HDB17_170904a_6.2'	2.78	33.08	19
'HDB17_170906a_6.3'	1.62	11.35	70
HDB17_170912a_6.1'	7.69	13.29	21
'HDB17_170928a_2.1'	4.36	36.47	86
'HDB17_170928a_4.1'	17.67	99.99	90
'HDB17_170928a_4.2'	8.05	14.62	16
'HDB17_171010a_2.1'	4.13	53.05	81
'HDB23_180221a_3.2'	28.38	39.83	15
'HDB23_180223a_3.1'	14.71	22.74	12
'HDB23_180223a_5.3'	4.26	22.80	66
'HDB34_190113a_7.1'	18.18	30.28	182
'HDB34_190115a_5.1'	16.53	40.53	15
'HDB34_190117a_4.1'	2.91	5.46	33
'HDB34_190118a_4.1'	4.25	8.39	97
'HDB34_190207a_8.1'	14.87	28.86	197
'HDB30_181002a_2.2'	2.85	8.52	95
HDB17_170811a_4.2'	11.62		
'HDB17_170812a_3.1'	23.68		
'HDB17_170912a_4.1'	35.32		
'HDB23_180225a_3.1'	16.24		
'HDB34_190115a_4.2'	4.73		
'HDB34_190123a_2.1'	53.69		
'HDB34_190127a_2.1'	18.92		
'HDB34_190207a_6.1'	17.97		
'HDB30_181002a_2.1'	5.26		
**Average ± standard error**	**15.32 ± 1.84**	**36.99 ± 5.24**	**77.0 ± 9.52**

Table S4. Baseline firing rate for all identified BFPVNs (n = 36) and punishment-evoked peak firing rate of punishment-activated BFPVNs (n = 27) along with peak latency of punishment response.
