# Supplementary material for: Parvalbumin-expressing basal forebrain neurons mediate learning from negative experience
Source: Nat Commun. 2024 Jun 7;15:4768. doi: 10.1038/s41467-024-48755-7 (PMC11161511; doi:10.1038/s41467-024-48755-7)
Supplement: Supplementary file 3 — Reporting Summary [file 41467_2024_48755_MOESM3_ESM.pdf]

nature portfolio

Corresponding author(s): Balázs Hangya

Last updated by author(s): Mar 13, 2024

# Reporting Summary

Nature Portfolio wishes to improve the reproducibility of the work that we publish. This form provides structure for consistency and transparency in reporting. For further information on Nature Portfolio policies, see our Editorial Policies and the Editorial Policy Checklist.

## Statistics

For all statistical analyses, confirm that the following items are present in the figure legend, table legend, main text, or Methods section.

| n/a | Confirmed | |
|---|---|---|
| ☐ | ☒ | The exact sample size (*n*) for each experimental group/condition, given as a discrete number and unit of measurement |
| ☒ | ☐ | A statement on whether measurements were taken from distinct samples or whether the same sample was measured repeatedly |
| ☐ | ☒ | The statistical test(s) used AND whether they are one- or two-sided<br>*Only common tests should be described solely by name; describe more complex techniques in the Methods section.* |
| ☒ | ☐ | A description of all covariates tested |
| ☒ | ☐ | A description of any assumptions or corrections, such as tests of normality and adjustment for multiple comparisons |
| ☐ | ☒ | A full description of the statistical parameters including central tendency (e.g. means) or other basic estimates (e.g. regression coefficient) AND variation (e.g. standard deviation) or associated estimates of uncertainty (e.g. confidence intervals) |
| ☐ | ☒ | For null hypothesis testing, the test statistic (e.g. *F*, *t*, *r*) with confidence intervals, effect sizes, degrees of freedom and *P* value noted<br>*Give P values as exact values whenever suitable.* |
| ☒ | ☐ | For Bayesian analysis, information on the choice of priors and Markov chain Monte Carlo settings |
| ☒ | ☐ | For hierarchical and complex designs, identification of the appropriate level for tests and full reporting of outcomes |
| ☒ | ☐ | Estimates of effect sizes (e.g. Cohen's *d*, Pearson's *r*), indicating how they were calculated |

*Our web collection on statistics for biologists contains articles on many of the points above.*

## Software and code

Policy information about availability of computer code

| | |
|---|---|
| Data collection | The Open Ephys 0.4.4.1 data acquisition system, and the Doric fiber photometry system was used to collect data. In vitro recordings were performed with a Multiclamp 700B amplifier (Molecular Devices) and pClamp 11 Software suite (Molecular Devices). |
| Data analysis | Data analysis was carried out using custom written Matlab code (R2016a, Mathworks). MATLAB codes generated for this study are available at https://github.com/hangyabalazs/PV_Pavlovian_analysis and also https://doi.org/10.5281/zenodo.10808341. |

For manuscripts utilizing custom algorithms or software that are central to the research but not yet described in published literature, software must be made available to editors and reviewers. We strongly encourage code deposition in a community repository (e.g. GitHub). See the Nature Portfolio guidelines for submitting code & software for further information.

## Data

Policy information about availability of data

All manuscripts must include a data availability statement. This statement should provide the following information, where applicable:

- Accession codes, unique identifiers, or web links for publicly available datasets
- A description of any restrictions on data availability
- For clinical datasets or third party data, please ensure that the statement adheres to our policy

The in vivo electrophysiology, fiber photometry and behavioral data generated in this study have been deposited at https://doi.org/10.6084/

m9.figshare.22776776.v1, https://doi.org/10.6084/m9.figshare.25403350 and https://doi.org/10.6084/m9.figshare.25403593. Source data are provided with this paper.

## Research involving human participants, their data, or biological material

Policy information about studies with human participants or human data. See also policy information about sex, gender (identity/presentation), and sexual orientation and race, ethnicity and racism.

| | |
|---|---|
| Reporting on sex and gender | No human research participants were involved in this study. |
| Reporting on race, ethnicity, or other socially relevant groupings | No human research participants were involved in this study. |
| Population characteristics | No human research participants were involved in this study. |
| Recruitment | No human research participants were involved in this study. |
| Ethics oversight | No human research participants were involved in this study. |

Note that full information on the approval of the study protocol must also be provided in the manuscript.

# Field-specific reporting

Please select the one below that is the best fit for your research. If you are not sure, read the appropriate sections before making your selection.

☒ Life sciences ☐ Behavioural & social sciences ☐ Ecological, evolutionary & environmental sciences

For a reference copy of the document with all sections, see nature.com/documents/nr-reporting-summary-flat.pdf

# Life sciences study design

All studies must disclose on these points even when the disclosure is negative.

| | |
|---|---|
| Sample size | Sample size was chosen based on other studies in the field (Hangya et al., 2015, Cohen et al., 2012, Legaria et al., 2022) |
| Data exclusions | In the ArchT experiments (Fig.4), 2 mice were excluded due to lack of viral expression. In the channelrhodopsin experiments (Fig. S4), 12 mice were excluded due to lack of viral expression. In the fiber photometry experiments, 1 SOM-IRES-Cre animal was exluded due to lack of viral expression and fluorescent signal. |
| Replication | All experiments were successfully replicated in multiple mice as indicated in the figures and the text. The fiber photometry experiments confirmed the results of the electrophysiology experiments. |
| Randomization | Mice were randomly assigned to treated and control groups for the optogenetic experiments. Mice were not allocated to groups in other experiments. |
| Blinding | The optogenetic experiment was blinded. Mice were not allocated to groups in other experiments. |

# Reporting for specific materials, systems and methods

We require information from authors about some types of materials, experimental systems and methods used in many studies. Here, indicate whether each material, system or method listed is relevant to your study. If you are not sure if a list item applies to your research, read the appropriate section before selecting a response.

### Materials & experimental systems

| n/a | Involved in the study | |
|---|---|---|
| ☐ | ☒ | Antibodies |
| ☒ | ☐ | Eukaryotic cell lines |
| ☒ | ☐ | Palaeontology and archaeology |
| ☐ | ☒ | Animals and other organisms |
| ☒ | ☐ | Clinical data |
| ☒ | ☐ | Dual use research of concern |
| ☒ | ☐ | Plants |

### Methods

| n/a | Involved in the study | |
|---|---|---|
| ☒ | ☐ | ChIP-seq |
| ☒ | ☐ | Flow cytometry |
| ☒ | ☐ | MRI-based neuroimaging |

## Antibodies

| | |
|---|---|
| Antibodies used | We used 9 primary and 7 secondary antibodies, listed in Tables S2 and S3. |
| Validation | Validation of the primary antibodies used are provided in Table S2. Secondary antibodies were validated by injecting them without the primaries and confirming the lack of staining. |

## Animals and other research organisms

Policy information about studies involving animals; ARRIVE guidelines recommended for reporting animal research, and Sex and Gender in Research

| | |
|---|---|
| Laboratory animals | Adult (over two months old) male Pvalb-IRES-Cre (PV-IRES-Cre or PV-Cre; n = 60, The Jackson Laboratory, RRID: IMSR_JAX:017320) and SOM-IRES-Cre (also known as Sst-IRES-Cre; n = 4, The Jackson Laboratory, RRID: IMSR_JAX:013044) were used for recording, optogenetic activation and inhibition, bulk calcium recording and for immunohistochemistry, and 30-60 days old PV-IRES-Cre mice were used for acute slice electrophysiology (n = 7, males). Animals were housed individually in 36 x 20 x 15 cm cages under a standard 12-hours light-dark cycle (lights on at 8 a.m.) with food available ad libitum. During behavioral training, mice were water-restricted to 1 ml per day. Temperature and humidity were kept at 21 ± 1 °C and 50-60%, respectively. |
| Wild animals | This study did not involve wild animals. |
| Reporting on sex | Male mice were used, because of their larger body size that allowed the toleration of chronic multichannel electrophysiology - fiber optic implants. |
| Field-collected samples | This study did not involve field-collected samples. |
| Ethics oversight | Experiments were carried out according to the regulations of the Hungarian Act of Animal Care and Experimentation (1998; XXVIII, section 243/1998, renewed in 40/2013) in accordance with the European Directive 86/609/CEE and modified according to the Directive 2010/63/EU. Experimental procedures were reviewed and approved by the Animal Welfare Committee of the Institute of Experimental Medicine, Budapest and by the Committee for Scientific Ethics of Animal Research of the National Food Chain Safety Office of Hungary (PE/EA/675-4/2016; PE/EA/1212-5/2017; PE/EA/864-7/2019). |

Note that full information on the approval of the study protocol must also be provided in the manuscript.

## Plants

| | |
|---|---|
| Seed stocks | No plants were involved in this study. |
| Novel plant genotypes | No plants were involved in this study. |
| Authentication | No plants were involved in this study. |
